# PRognostic factor of Early Death In phase II Trials or the end of ‘sufficient life expectancy’ as an inclusion criterion? (PREDIT model)

**DOI:** 10.1186/s12885-016-2819-7

**Published:** 2016-10-04

**Authors:** Thomas Grellety, Sophie Cousin, Louis Letinier, Pauline Bosco-Lévy, Stéphanie Hoppe, Damien Joly, Nicolas Penel, Simone Mathoulin-Pelissier, Antoine Italiano

**Affiliations:** 1 Department of Medical Oncology, Institut Bergonié, Comprehensive Cancer Centre Bordeaux, 229 cours de l’Argonne, 33076 Bordeaux, France; 2 University of Bordeaux, Bordeaux, France; 3 Clinical and Epidemiological Research Unit Institut Bergonié, Bordeaux, France; 4 General Oncology Department, Centre Oscar Lambret, Lille, France; 5 INSERM, CIC1401 Epidemiological unit, Institut Bergonié, Bordeaux, France

**Keywords:** Phase II trial, Early death, Prognostic factors, “life expectancy” criterion, Drug trials

## Abstract

**Background:**

Optimizing patient selection is a necessary step to design better clinical trials. ‘Life expectancy’ is a frequent inclusion criterion in phase II trial protocols, a measure that is subjective and often difficult to estimate. The aim of this study was to identify factors associated with early death in patients included in phase II studies.

**Methods:**

We retrospectively collected medical records of patients with advanced solid tumors included in phase II trials in two French Comprehensive Cancer Centers (Bordeaux, Center 1 set; Lille, Center 2 set). We analyzed patients’ baseline characteristics. Predictive factors associated with early death (mortality at 3 months) were identified by logistic regression. We built a model (PREDIT, PRognostic factor of Early Death In phase II Trials) based on prognostic factors isolated from the final multivariate model.

**Results:**

Center 1 and 2 sets included 303 and 227 patients, respectively. Patients from Center 1 and 2 sets differed in tumor site, urological (26 % vs 15 %) and gastrointestinal (18 % vs 28 %) and in lung metastasis incidence (10 % vs 49 %). Overall survival (OS) at 3 months was 88 % (95 % CI [83.5; 91.0], Center 1 set) and 91 % (95 % CI [86.7; 94.2], Center 2 set). Presence of a ‘life expectancy’ inclusion criterion did not improve the 3-month OS (HR 0.6, 95 % CI [0.2; 1.2], *p* = 0.2325). Independent factors of early death were an ECOG score of 2 (OR 13.3, 95%CI [4.1; 43.4]), hyperleukocytosis (OR 5.5, 95 % CI [1.9; 16.3]) and anemia (OR 2.8, 95 % CI [1.1; 7.1]). Same predictive factors but with different association levels were found in the Center 2 set. Using the Center 1 set, ROC analysis shows a good discrimination to predict early death (AUC: 0.89 at 3 months and 0.86 at 6 months).

**Conclusions:**

Risk modeling in two independent cancer populations based on simple clinical parameters showed that baseline ECOG of 2, hyperleukocytosis and anemia are strong early-death predictive factors. This model allows identifying patients who may not benefit from a phase II trial investigational drug and may, therefore, represent a helpful tool to select patients for phase II trial entry.

**Electronic supplementary material:**

The online version of this article (doi:10.1186/s12885-016-2819-7) contains supplementary material, which is available to authorized users.

## Background

Phase II trials in oncology are an essential part in anti-cancer drug development as they provide relevant data regarding toxicity and proof of efficacy. These assessments are necessary to make the ‘go or no-go’ decision before starting large controlled randomized phase III trials [1]. In oncology, there are more phase II (45 % vs 23 %) but fewer phase III (13 % vs 23 %) trials than in other specialties [2]. Phase II to phase III represents the riskiest transition point of the drug development pathway [3, 4], as proven by the very high attrition rate between a successful phase II and the subsequent phase III trial. Enhancing the overall quality of phase II trials is therefore critical for drug development, and could benefit from changes at several levels, from the use of randomization in the study design [5] to the improvement in the quality of publication [6]. Furthermore, there is a need to rethink the selection of large numbers of patients for phase II trials that raise ethical and cost questions. Indeed, patient selection has been recognized as being of upmost importance in the design of clinical trials [7]. Although many efforts have been made in phase I trials wherein a careful patient selection likely increases the benefit of the trial to patients, no such initiative has been taken for phase II trials. Similarly, there is an increase in the average number of inclusion criteria for phase II trials, such as ‘sufficient life expectancy’ at screening [8]. Life expectancy is difficult to estimate in clinical practice and depends on the physician’s consideration, making it not only irreproducible but also insufficient to predict any benefit for the patient, as most patients enroll with a hope for therapeutic benefit [9]. Ethical consideration should therefore lead physicians to include patients only in cases of potential benefit from the investigational drug. This would require identifying those patients that would survive long enough for the investigational treatment to be effective. Despite the crucial role of phase II trials in drug development, no tool has been published that allows a better selection of patients based on their prognostic. The aim of this pilot study is to develop a model to identify prognostic factors of early death in adult cancer patients included in oncology phase II trials based on two sets of patients from two French Comprehensive Cancer Centers. Relevant prognostic factors will help investigators identify participants unsuitable for such studies.

## Methods

### Selection of patients

The first patient set (Center 1 set) included all patients involved in phase II clinical trials at the Institut Bergonié, Comprehensive Cancer Center (Bordeaux, France), between January 2008 and December 2012. We selected all trials investigating anticancer drugs and having included adults (aged 18 or older) with advanced or metastatic solid tumors. Trials investigating supportive care, surgical procedures or radiotherapy were excluded. Patients had received at least one dose of the investigational agent. The second set (Centre 2 set) was from the Oscar Lambret Cancer Center (Lille, France), with all patients included in a phase II clinical trial between January 2011 and July 2014 that met the same criteria.

For each patient, retrospective baseline data were recorded at inclusion in the phase II trial: age, gender, body mass index (BMI), ECOG performance status, histology, number and sites of metastasis, treatment type, biological data (serum albumin, Lactate Dehydrogenase (LDH), platelets, leukocyte and lymphocyte counts, hemoglobin level, sodium, potassium and calcium level, alkaline phosphatase, alanine and aspartate transaminase and c-reactive protein). Furthermore, for each patient we recorded the date of inclusion, and date and cause of study withdrawal.

The following data regarding the design of the clinical trials were extracted from each protocol: presence of a “life expectancy” inclusion criterion, randomized trial (Yes vs No), number of previous treatment lines authorized and nature of the promoter (academic vs industrial). Study data were collected and managed using REDCap electronic data capture tools [10].

### Statistical methods

Variables were described using median, mean and extreme values. Categorical variables were classified based on the normal values (for biological variables, BMI). Biological variables were classified as normal, below normal and above normal. Overall survival (OS) was defined as the time from inclusion in a trial to death from any cause. Patients lost during follow-up were censored at their last visit. Survival was estimated using the Kaplan–Meier method. For our main analysis, we used early deaths, defined as all deaths occurring up to 3 months from inclusion. We also performed a secondary analysis for deaths occurring up to 6 months from inclusion. Three- and six-month’ cut-off’s were chosen due to their discriminant nature in the detection of prognostic factors. Three months represents the classical cut-off point for the first evaluation of safety and efficacy in clinical trials. It has commonly been used in studies of prognostic factors for patients included in phase I trials [11, 12] and is relevant regarding the median overall survival of 9.4 months for patients included in phase II trials, as published in a recent meta-analysis by Schwaederle M. et al. [13].

On the Center 1 set, we performed a logistic model to estimate odds ratio (OR) and 95 % confidence interval (95 % CI) of the association between early death and clinical or biological variables. All variables associated with a significantly increased risk of early death (*p* < 0.05) were considered for multivariate analysis. Variables such as age, sex and tumor localization were included in all models due to clinical relevance. Selection of variables for the multivariate model was performed following a step-by-step forward strategy. In order to limit the number of variables in the final multivariate model, clinical and laboratory variables were first selected in two separate specific multivariate models using stepwise logistic approach. Each clinical and biological variable selected in their respective multivariate model was entered into a third and final model before adjusting for age, sex and tumor localization. The threshold of 0.05 for statistical significance was used to maintain the variable in the model. The stringent alpha level allowed limiting the selection to those factors that are relevant from a clinician’s perspective.

A model (PREDIT, PRognostic factor of Early Death In phase II Trial) was built with the prognostic factors isolated from the final multivariate model in the Center 1 set. Adequacy was established using the Hosmer & Lemeshow test. [14]. Discrimination of mortality at 3 and 6 months was evaluated using the receiver operator characteristic area under the curve (AUC). Finally, we performed the same analyses in the Centre 2 set. Statistical analyses were carried out using the SAS software, version 9.3 (SAS Institute, Inc., Cary, NC).

## Results

### Characteristics of the trials

Fifty-one trials were included for analysis in the Center 1 set and 40 in the Center 2 set, with recruitment ranging from one to 31 patients. Patient characteristics are described in Table 1. Twenty-six trials (51 %) in the Center 1 set and 27 trials in the Center 2 set (68 %) were sponsored by a pharmaceutical company. Most phase II trials were randomized (59 % in the Center 1 set and 63 % in the Center 2 set). Treatments differed between the two sets: whereas the ratio between targeted therapy (149, 49 %) and chemotherapy (154, 51 %) was well-balanced in the Center 1 set, chemotherapy was more frequently used (157, 69 %) in the Center 2 set.

**Table 1:** Characteristics of trials and outcomes for Center 1 and Center 2 sets

Characteristics	Center 1 Set (*N* = 303)		Center 2 Set (*N* = 227)		*P* Chi2
	*N* (%)	Median (Min-Max)	*N* (%)	Median (Min-Max)	
Number of trials	51		40		
Patients by trials		4 (1–31)		2 (1–22)	
Trial randomization					0.13
Yes	30 (59)		25 (63)		
No	21 (41)		15 (37)		
Trial promotion					0.08
Academic	25 (49)		27 (68)		
Industrial	26 (51)		13 (32)		
Protocol defined treatments					<0.001
Chemotherapy-based regimen	154 (51)		157 (69)		
Targeted therapies only (targeted therapies and/or endocrine therapy)	149 (49)		70 (31)		
Protocol specified “life expectancy” criterion (per patient)					0.003
Yes	73 (24)		82 (36)		
No	230 (76)		145 (64)		
Treatment duration (months)		4 (0–44)		4 (0–49)	
Reason for discontinuing trial					<0.001
Progression	198 (65)		136 (60)		
Programmed end of the trial	42 (14)		6 (3)		
Toxicity	32 (10)		37 (16)		
Death	12 (4)		2 (1)		
Patient Retrial	8 (3)		9 (4)		
Other	8 (3)		18 (8)		
NA	3 (1)		19 (8)		
Median follow-up (months)		17 (0–77)		12 (0–50)	
Three-month mortality rate	37 (12)		20 (9)		
Six-month mortality rate	59 (20)		41 (18)		

*Abreviations: NA* not available

### Patient characteristics

The Center 1 and Center 2 sets included 303 and 227 patients, respectively. Median age was 62 years (Interquartile Range, 19) in the Center 1 set and 60 (Interquartile Range, 23) years in the Center 2 set. The male to female ratio was similar in both sets (Center 1 set: 1.5 and Center 2 set: 1.4). Primary tumor sites were equally distributed for sarcomas (123, 41 %; 91, 40 %) and breast (29, 10 %; 20, 9 %) but differed significantly for urological (78, 26 %; 35, 15 %) and gastrointestinal (54, 19 %; 63, 28 %) cancers. There were 241 (80 %) and 183 (81 %) patients with two or less metastatic sites in the Center 1 and Center 2 sets, respectively. Occurrence of liver metastases was similar in both groups, (Center 1 set: 106, 35 %; Center 2 set: 73, 32 %) whereas lung metastases were rarer in the Center 1 set (Center 1 set: 30, 10 %; Center 2 set: 111, 49 %); there was more bone and extra-regional lymph nodes involvement in the Center 1 set. Median number of previous lines of treatment was one (Center 1 set: range 0–4; Center set: range 0–6). Clinical and biological values at baseline are described in Table 2.

**Table 2:** Clinical and biological characteristics at baseline for Center 1 and Center 2 sets

Characteristics at baseline	Center 1 Set (*N* = 303)		Center 2 Set (*N* = 227)		*P* Chi2
	*N* (%)	Median (Min-Max)	*N* (%)	Median (Min-Max)	
Sex					0.64
Male	181 (60)		131 (58)		
Female	122 (40)		96 (42)		
Age		62 (IQR, 19)		60 (IQR, 23)	0.07
< 50	55 (18)		42 (19)		
50–65	116 (38)		107 (47)		
≥ 65	132 (44)		78 (34)		
Performance status					0.82
ECOG 0–1	278 (92)		207 (91)		
ECOG 2	25 (8)		20 (9)		
BMI (kg/m^2^)		25 (16–39)		26 (16–45)	-^a^
< 18.5	13 (4)		9 (4)		
18.5–25	147 (49)		83 (37)		
≥ 25	143 (47)		107 (47)		
NA	0		28 (12)		
Previous treatments		1 (0–4)		1 (0–6)	<0.001
0	9 (3)		106 (47)		
1	185 (61)		67 (30)		
2	74 (24)		32 (14)		
3	27 (9)		19 (8)		
4 and more	8 (3)		3 (1)		
Cancer localization					0.01
Sarcomas	123 (40)		91 (40)		
Urological	78 (26)		35 (15)		
Gastrointestinal	54 (18)		63 (28)		
Breast	29 (10)		20 (9)		
Other	19 (6)		18 (8)		
Involved areas					0.55
Loco-regional disease	18 (6)		19 (8)		
1–2 metastatic sites	223 (74)		164 (72)		
≥ 3 metastatic sites	62 (20)		44 (19)		
Hemoglobin level					0.08
< Normal	106 (35)		94 (41)		
Normal	196 (65)		126 (56)		
NA	1 (0.3)		7 (3)		
Leukocyte level					0.18
< Normal	14 (5)		15 (6)		
Normal	255 (84)		171 (75)		
> Normal	33 (11)		33 (15)		
NA	1 (0.3)		8 (4)		
Albumin level					-^a^
< Normal	25 (8)		87 (38)		
Normal	236 (78)		111 (50)		
NA	32 (11)		28 (12)		
LDH level					-^a^
< Normal	133 (44)		137 (60)		
Normal	55 (18)		51 (23)		
NA	111 (37)		34 (15)		

*Abbreviations: BMI* body mass index, *ECOG* Eastern Cooperative Oncology Group, *IQR* interquartile range, *LDH* lactate dehydrogenase, *NA* not available

^a^Due to a non-applicable rate of 10 % or more, BMI, Albumin level and LDH level variables cannot be tested by the chi test

### General description

**Fig. 1:**
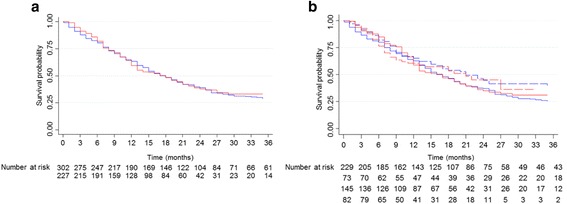
Overall survival in the two sets. **a** Overall survival in the Center 1 set (*blue*) and Center 2 set (*red*). **b** Survival depending on the presence (*dotted lines*) or absence (*full lines*) of ‘life expectancy’ criterion for each patient included regarding the respective trial’s protocol, in the Center 1set (*blue*) and Center 2 set (*red*)

Median time to trial discontinuation was 4.0 months in the two sets (range 0–44 and 0–49, respectively). Most patients were withdrawn from the study due to disease progression (Center 1 set: 198, 65 %; Center 2 set: 136, 60 %) or to toxicity (Center 1 set: 32, 11 %; Center 2 set: 37, 16 %). Overall survival at 3 months in the Center 1 and 2 sets were 88 % (95 % CI, 83.5–91.0) and 91 % (95 % CI, 86.7–94.2), respectively. Life expectancy was included as an eligibility criterion in 13 trials (73 patients, 24 %) in the Center 1 set and 19 trials (82 patients, 36 %) in the Center 2 set. The presence of life expectancy among the inclusion criteria did not improve the 3-month OS in either the Center 1 (hazards ratio [HR] 0.6, 95 % CI, 0.2–1.2, *P* = .23) or the Center 2 (HR 0.7, 95 % CI, 0.3–2.0, *P* = .55) set (Fig. 1).

### Factors associated with 3-month early deaths

Results from univariate and multivariate analyses are presented in Table 3 and Additional file 1: Table S1 (online-only supplementary material). Factors associated with the 3-month mortality in multivariate analysis in the Center 1 set, after adjustment on age, sex and tumor localization, included an ECOG performance status of 2 (OR 13.3, 95 % CI, 4.1–43.4), hyperleukocytosis (OR 5.5, 95 % CI, 1.9–16.3) and anemia (OR 2.8, 95 % CI, 1.1–7.1). Based on these three factors, we calculated a risk (PREDIT model) for patients included in a phase II trial with the following equation:

**Table 3:** Factors associated with early death at 3 months in the Center 1 set (*N* = 303)

		Univariate analysis			Multivariate analysis		
Characteristics at baseline	Early deaths *N* (%)	OR	95 % CI	*P*	OR	95 % CI	*P*
Age				**0.01**			0.21
< 50	12 (32)	1			1		
[50–65]	17 (46)	0.2	[0.1–0.6]		1.8	[0.6–5.7]	
≥65	8 (22)	0.6	[0.3–1.4]		0.7	[0.2–2.4]	
Sex				0.17			0.1500
Female	11 (30)	1			1		
Male	26 (70)	1.7	[0.8–3.5]		2.2	[0.8–6.2]	
Performance status				**<0.001**			**<0.001**
ECOG 0/1	21 (57)	1			1		
ECOG 2	16 (43)	21.7	[8.5–54.9]		13.3	[4.1–43.4]	
Cancer localization				**0.02**			0.08
Sarcoma	26 (70)	1			1		
Gastrointestinal	4 (11)	0.3	[0.1–0.9]		0.2	[0.0–0.7]	
Urological	5 (14)	0.3	[0.1–0.7]		0.3	[0.1–1.2]	
Breast	2 (5)	0.3	[0.1–1.2]		0.4	[0.1–1.0]	
Other	0	<0.1	[<0.1– > 999]		<0.1	[<0.1– > 999]	
Hemoglobin level				**<0.001**			**0.03**
Normal	12 (32)	1			1		
< Normal	25 (68)	4.8	[2.3–10.0]		2.8	[1.1–7.1]	
Leukocyte level				**<0.001**			**0.006**
Normal	22 (59)	1			1		
< Normal	1 (3)	0.8	[0.1–6.5]		0.4	[0.0–5.4]	
> Normal	14 (38)	7.8	[3.4–17.6]		5.5	[1.9–16.3]	

*Abbreviations: CI* confidence interval, *ECOG* Eastern Cooperative Oncology Group, *OR* odds ratio

Bold data reflects significant value

*Model = 0.4177 x (Age = [50–65[) + 0.5950 x (Age = ≥ 65) + 0.7703 x (Sex = Male) - 0.8658 x (Cancer localization = Breast) - 1.3116 x (Cancer localization = Urogenital) - 13.3383 x (Cancer localization = Other) - 1.7239 x (Cancer localization = Gastrointestinal) + 2.5882 x (ECOG = 2) - 0.8887 x (Leukocyte level = Below the norm) + 1.7050 x (Leukocyte level = Above the norm) + 1.0323x (Hemoglobin level = Below the norm)*

Risk calculation revealed a good predictive value for both 3-month and 6-month mortality rates, with AUC values ranging from 0.7 to 0.9 in both sets. More concretely, in the overall population, patients with 0, 1, 2 and 3 risk factors had a rate of a 3-month early-death of 2 % (7/292), 14 % (24/175), 38 % (20/53) and 60 % (6/10) and a rate of 6-month early-death of 7 % (19/292), 28 % (49/175), 47 % (25/53) and 70 % (7/10), respectively.

## Discussion

Phase II trials are crucial screening tools to assess whether an anti-cancer drug has sufficient activity to warrant further investigation in large, costly phase III trials. In this respect, patients should be selected for such trials in a manner that maximizes the potential to assess the clinical activity of the investigational drug. Patients in poor conditions and with a limited life expectancy are not likely to derive significant benefit from the investigational therapy and inclusion of such patients may preclude valid conclusions about the clinical activity of the drug. Therefore, appropriate assessment of life expectancy is crucial to avoid inclusion of patients who are at higher risk of early death and who have low probability of clinical benefit. Our prognostic factors, ECOG performance status of 2, hyperleukocytosis and anemia, validated in two independent sets, could provide physicians with an objective tool to help in this assessment.

Performance status is a well-known bad prognostic factor in oncology, associated with early death and already described for patients included in phase I trials [15, 16]. Anemia is very frequent in oncology with a prevalence of 39 % at onset of cancer, and 68 % of patients present anemia at least once in the subsequent 6 months [17]. It leads to an overall relative increase in risk of death of 65 % (54–77 %) [18] that can be related to various factors. A decrease in WHO performance score and quality of life are well-known consequences [17] that can limit options for specific cancer treatments. The worst outcomes associated to anemia can also be linked to its biological consequences. As an example, a more aggressive cancer biology is connected to the promotion of hypoxia-inducible factor 1, which is induced by anemic hypoxia and has been described as a tumor metastasis enhancer [19]. Initial hyperleukocytosis (often associated with neutrophilia) is a frequent event in patients with solid tumors, with an incidence ranging from 4 to 26 % [20], and has been associated with poor outcome in several solid tumor types [21–28]. Indeed, leukocytosis is related to tumor burden [27, 29]. Additionally, it can be a consequence of processes such as infection and/or inflammation or corticosteroids treatment [29, 30], which can themselves be of bad prognostic.

This preliminary study allowed developing a first model that needs to be validated at the national level. One of the advantages of a two-step procedure is the possibility to evaluate the feasibility on the data collection in the medical record. One of the disadvantages is that preliminary results are not confirmed on a larger population. Besides the limited power, the main limitation of our study lies in its retrospective nature. Prospectively defining a model for patients that would be included in a phase II trial would be very complex, and, as a consequence, only patients who passed screening and received at least one dose of the investigational agent were included in the study (whatever the set). One of the strengths of our study is that the sets originated from two different cancer centers. We identified the same three prognostic factors in the Center 2 set despite differences in the characteristics of the two populations. This demonstrates a strong relevance of these factors to predict early death regardless of the heterogeneity of patients, primary tumor sites, treatments and management strategies. Further work will aim at validating the model in an independent and wider cohort of patients such as a national cancer registry.

## Conclusions

Risk modeling based on simple clinical parameters including hemoglobin level, leukocyte count and ECOG performance status indicated that patients with two or more prognostic factors had a significant risk of early death. Our results clearly suggest that these patients should be considered carefully for inclusion in a phase II clinical trial. Our model may represent a helpful tool in the process of patient selection for phase II trial entry.

## Additional file

Additional file 1: Table S1. Univariate analysis of factors non-associated with 3-month mortality in the multivariate model for the Centre 1 set. (DOCX 25 kb)

## Abbreviations

AUC: Area under the curve
BMI: Body mass index
CI: Confidence interval
ECOG: Eastern cooperative oncology group
HR: Hazards ratio
LDH: Lactate dehydrogenase
NA: Not available
OR: Odds ratio
OS: Overall survival
