# Supplementary material for: PRognostic factor of Early Death In phase II Trials or the end of ‘sufficient life expectancy’ as an inclusion criterion? (PREDIT model)
Source: BMC Cancer. 2016 Oct 4;16:768. doi: 10.1186/s12885-016-2819-7 (PMC5050995; doi:10.1186/s12885-016-2819-7)
Supplement: Additional file 1: Table S1. — Univariate analysis of factors non-associated with 3-month mortality in the multivariate model for the Centre 1 set. (DOCX 25 kb) [file 12885_2016_2819_MOESM1_ESM.docx]

**Table S1.** Univariate analysis of factors non-associated with 3-month mortality in the multivariate model for the Centre 1 set.

| **Characteristics at baseline** | **Early deaths**  **N (%)** | **OR** | **95% CI** | ***P*** |
| --- | --- | --- | --- | --- |
| **BMI kg/m^2^** |  |  |  | **0.004** |
| < 18.5 | 5 (14) | 1 |  |  |
| [18.5-25[ | 22 (60) | 0.3 | [0.1-1.0] |  |
| ≥ 25 | 10 (27) | 0.1 | [0.0-0.4] |  |
| **Involved areas** |  |  |  | **0.02** |
| ≤ 2 | 24 (65) | 1 |  |  |
| > 2 | 13 (35) | 2.4 | [1.1-5.0] |  |
| **Liver Metastases** |  |  |  | 0.14 |
| No | 20 (54) | 1 |  |  |
| Yes | 17 (46) | 1.7 | [0.8-3.4] |  |
| **Lung Metastases** |  |  |  | 0.18 |
| No | 31 (84) | 1 |  |  |
| Yes | 6 (16) | 1.9 | [0.7-5.1] |  |
| **Bone Metastases** |  |  |  | 0.59 |
| No | 25 (68) | 1 |  |  |
| Yes | 12 (32) | 0.8 | [0.4-1.7] |  |
| **Lymph nodes Metastases** |  |  |  | 0.67 |
| No | 23 (62) | 1 |  |  |
| Yes | 14 (38) | 0.9 | [0.4-1.7] |  |
| **Lymphocyte count** |  |  |  | **0.03** |
| Normal | 17 (46) | 1 |  |  |
| < Normal | 20 (54) | 2.1 | [1.1-4.3] |  |
| **Platelet count** |  |  |  | 0.08 |
| Normal | 27 (73) | 1 |  |  |
| < Normal | 5 (14) | 3.2 | [1.1 - 9.8] |  |
| > Normal | 5 (14) | 1.8 | [0.6 - 5.0] |  |
| **Sodium count** |  |  |  | **0.02** |
| Normal | 27 (73) | 1 |  |  |
| < Normal | 10 (27) | 3.2 | [1.4-7.3] |  |
| > Normal | 0 | < 0.1 | [< 0.1- > 999] |  |
| **Potassium count** |  |  |  | 0.08 |
| Normal | 31 (84) | 1 |  |  |
| < Normal | 4 (11) | 3.2 | [0.9-10.7] |  |
| > Normal | 2 (5) | 3.2 | [0.6-17.1] |  |
| **Calcium count** |  |  |  | **< 0.001** |
| Normal | 24 (67) | 1 |  |  |
| < Normal | 9 (25) | 11.5 | [3.9-33.7] |  |
| > Normal | 3 (8) | 0.8 | [0.2-3.0] |  |
| **Transaminase count** |  |  |  | **0.001** |
| Normal | 22 (60) | 1 |  |  |
| > Normal | 15 (41) | 3.4 | [1.6-7.4] |  |
| **PAL count** |  |  |  | **< 0.001** |
| Normal | 16 (43) |  |  |  |
| > Normal | 21 (57) | 3.7 | [1.7-8.0] |  |

**Abbreviations**: BMI: Body mass index; PAL: Phosphatase alkaline
